# Diagnosis and treatment of Robert’s uterus combined with transverse uterine septum: a case report and review of the literature

**DOI:** 10.3389/fmed.2024.1434957

**Published:** 2024-08-02

**Authors:** Mingqian Chen, Xiaoyu Yang, Bin Zhang, Jialiang Fu, Yi Lan, Yugang Chi

**Affiliations:** ^1^Department of Obstetrics and Gynecology, Women and Children's Hospital of Chongqing Medical University (Chongqing Health Center for Women and Children), Chongqing, China; ^2^Department of Ultrasound, Women and Children's Hospital of Chongqing Medical University (Chongqing Health Center for Women and Children), Chongqing, China; ^3^Department of Radiology, Women and Children's Hospital of Chongqing Medical University (Chongqing Health Center for Women and Children), Chongqing, China

**Keywords:** Robert’s uterus, transverse uterine septum, asymmetric septate uterus, uterine malformation, hysteroscopic metroplasty

## Abstract

**Background:**

Robert’s uterus combined with transverse uterine septum is a rare uterine malformation. Only one case has been reported to date, and it is challenging to diagnose and treat.

**Case presentation:**

Here we report the case of a 19-year-old female had undergone multiple treatments at a previous hospital for primary amenorrhea and periodic lower abdominal pain, with the cause remaining unclear. Ultrasonography and magnetic resonance imaging conducted at our hospital displayed the presence of uterine dysplasia with an incomplete septum. Following a multidisciplinary discussion, a preliminary diagnosis of Robert’s uterus combined with transverse uterine septum was made. Following this, laparoscopic exploration and ultrasound-guided hysteroscopic metroplasty were performed. The patient experienced periodic menstruation postoperatively and did not manifest periodic lower abdominal pain.

**Conclusion:**

Robert’s uterus combined with transverse uterine septum is a very rare uterine malformation, with hysteroscopic metroplasty being the preferred surgical option. Nevertheless, preoperative diagnosis is extremely difficult, and there are also many difficulties in the surgical process. This case outlines the diagnostic and treatment process of a patient with Robert’s uterus and transverse uterine septum. It is of great significance to fill the gap in clinical diagnosis and treatment of this special uterine malformation.

## Introduction

Robert’s uterus (asymmetric septate uterus) is a rare complex Müllerian malformation first described by French gynecologists in 1970 with an incidence <3% (1, 2). Indeed, only a few cases have been reported. Robert’s uterus is anatomically characterized by asymmetric uterine separation with unilateral menstrual blood retention in the uterus. This separation extends from the fundus to the upper part of the internal opening of the cervix, inclining to one side of the uterine cavity, and completely closes the uterine cavity on the ipsilateral side. This results in a blind hemicavity that does not communicate with the contralateral uterine cavity and vagina. On the other hand, the blind hemicavity is connected with the ipsilateral side of the fallopian tube. Notwithstanding, the uterine silhouette is unremarkable (3, 4). The lack of communication between the two uterine hemicavities leads to menstrual blood retention and reflux, hematometra, ipsilateral hematosalpinx, severe dysmenorrhea, pelvic endometriosis, and secondary reproductive dysfunction, encompassing infertility, abortion, premature delivery, and malpresentation-induced dystocia (5). Of note, only one case of Robert’s uterus combined with a transverse uterine septum was documented in 2017 (6).

Patients with Robert’s uterus combined with transverse septum do not discharge menstrual blood due to the complete obstruction of the reproductive tract, thereby exacerbating dysmenorrhea, which can be detected via Ultrasonography and magnetic resonance imaging (MRI) examinations (7). Ascribed to the rarity of this type of uterine malformation, it is susceptible to misdiagnosis as other types of uterine malformations, such as unicornuate uterus. Selecting an inadequate surgical approach may exert unfavorable effects on the patient’s future fertility (8). At present, there is no standardized surgical plan recommended for the management of Robert’s uterus combined with a transverse uterine septum. This case report outlines the diagnosis and treatment of Robert’s uterus combined with transverse uterine septum and intrauterine adhesion.

## Case presentation

**Figure 1:**
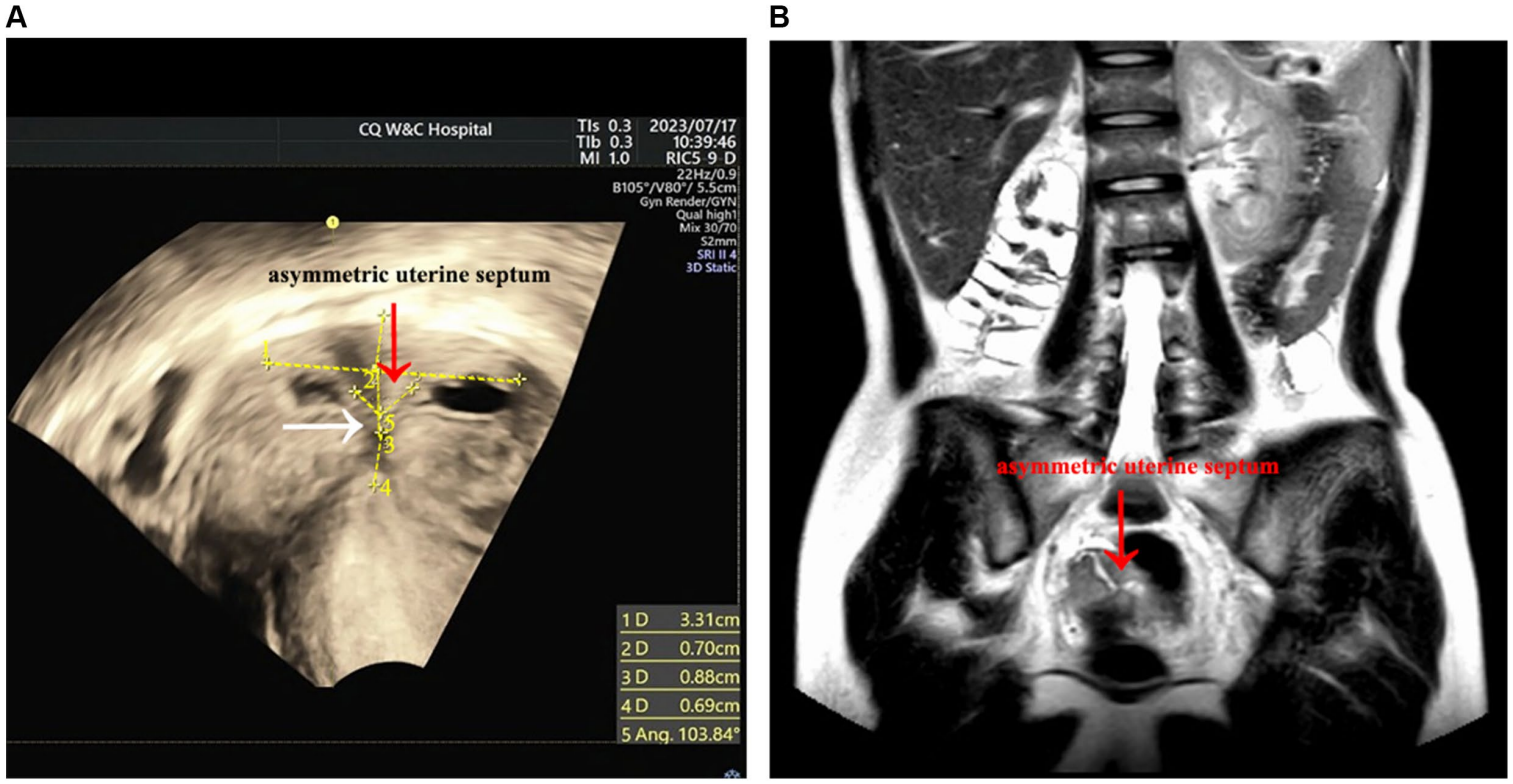
Preoperative imaging examination. **(A)** The red arrow points to the asymmetrical uterine septum, while the white arrow points to the obstruction site, i.e., the transverse uterine septum. However, the ultrasound was not completely clear, and the transverse uterine septum was not visualized. **(B)** The uterine fundus, indicated by the arrow, is depressed downward toward the uterine septum. Given that the thickness of the transverse uterine septum was below the MRI slice gap, the MRI image did not capture the transverse uterine septum.

The patient, a 19-year-old female with no sexual history, has been experiencing periodic lower abdominal pain since the age of 16 years. After many visits to local hospitals, no obvious abnormality was found in female hormone. Multiple ultrasound only showed adnexal mass, small uterus, no uterine malformation. The patient underwent progesterone testing and artificial cycle treatment, both displaying the absence of menstrual flow and no significant improvement in abdominal pain. While the periodic abdominal pain was relieved in January 2023, she did not menstruate. Afterward, the patient attended our hospital for further treatment. The gynecological vulvar examination was unremarkable, with visible hymen holes visible. Anal examination exposed that the right index finger could be extended into the rectum, allowing the visualization of part of the posterior vaginal wall at the margin of the hymen. At 7 cm into the rectum, the cervical canal was palpable, and no abnormal mass was detected. Review transrectal three-dimensional ultrasound (3D US) (GE Voluson E8, America) (Figure 1A) depicted that the horizontal transverse section of the uterine fundus revealed a hypoechoic separation between the two endometria, with a distance of roughly 3.3 cm between the bilateral uterine horns. In addition, the midpoint was approximately 0.7 cm away from the serosal layer of the uterine fundus, with a downward depression of about 0.9 cm and about 0.7 cm away from the internal cervix opening. Moreover, the angle between the bilateral uterine horns and the lowest point of the depression was 103.84°. An echoless area was detected in both uterine cavities. Diagnostic considerations: 1. Uterine malformation (consistent with sonographic changes of incomplete septate uterine); 2. Biadnexal cystic mass; 3. Bilateral horn strip echo zone: The possibility of fluid accumulation in the interstitial area of the fallopian tube was considered. MRI (Figure 1B) images delineated that the size of the uterus was about 4.7 × 3.7 × 7.0 cm, with a uterine body-to-cervical diameter ratio of about 3/4. The myometrium at the fundus of the uterus was locally convex toward the uterine cavity. In addition, the volume of the right kidney was significantly reduced to about 3.0 × 2.0 × 3.6 cm. Diagnostic considerations: 1. uterine malformation: possible uterine dysplasia with incomplete septum; 2. Right kidney dysplasia with multiple cysts; 3. Cystic space occupying bilateral adnexal areas, benign lesions, multiple cysts, and cystadenoma were considered; 4. Blood or fluid accumulation in the uterine cavity and bilateral fallopian tubes. Lastly, the results of the thyroid function, liver and kidney function, and coagulation function tests were normal. Following a multidisciplinary discussion, a preliminary diagnosis of Robert’s uterus combined with transverse uterine septum was made.

**Figure 2:**
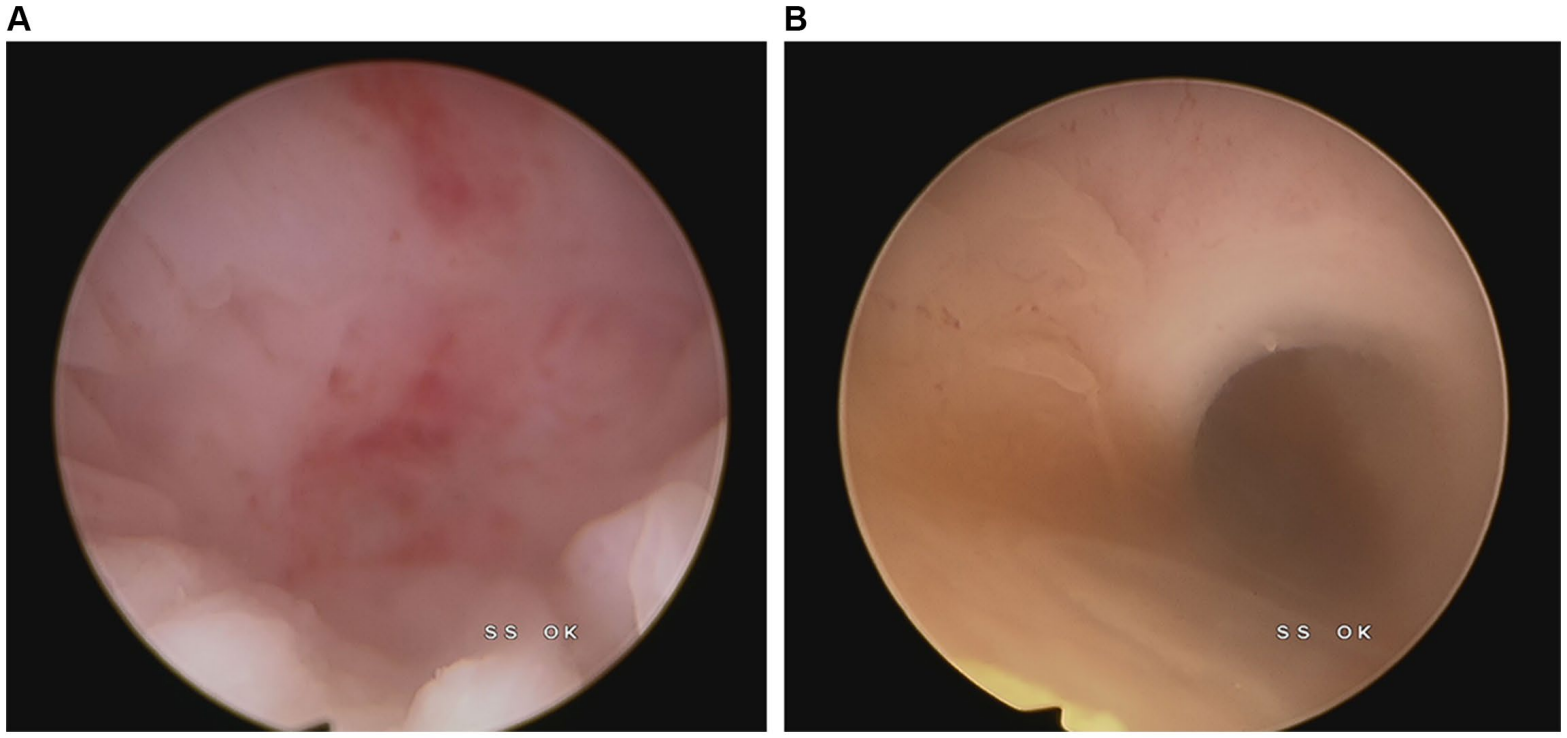
Cervical image during hysteroscopy. **(A)** The internal opening of the cervix is closed. **(B)** Brown menstrual blood outflow after puncturing the transverse uterine septum.

**Figure 3:**
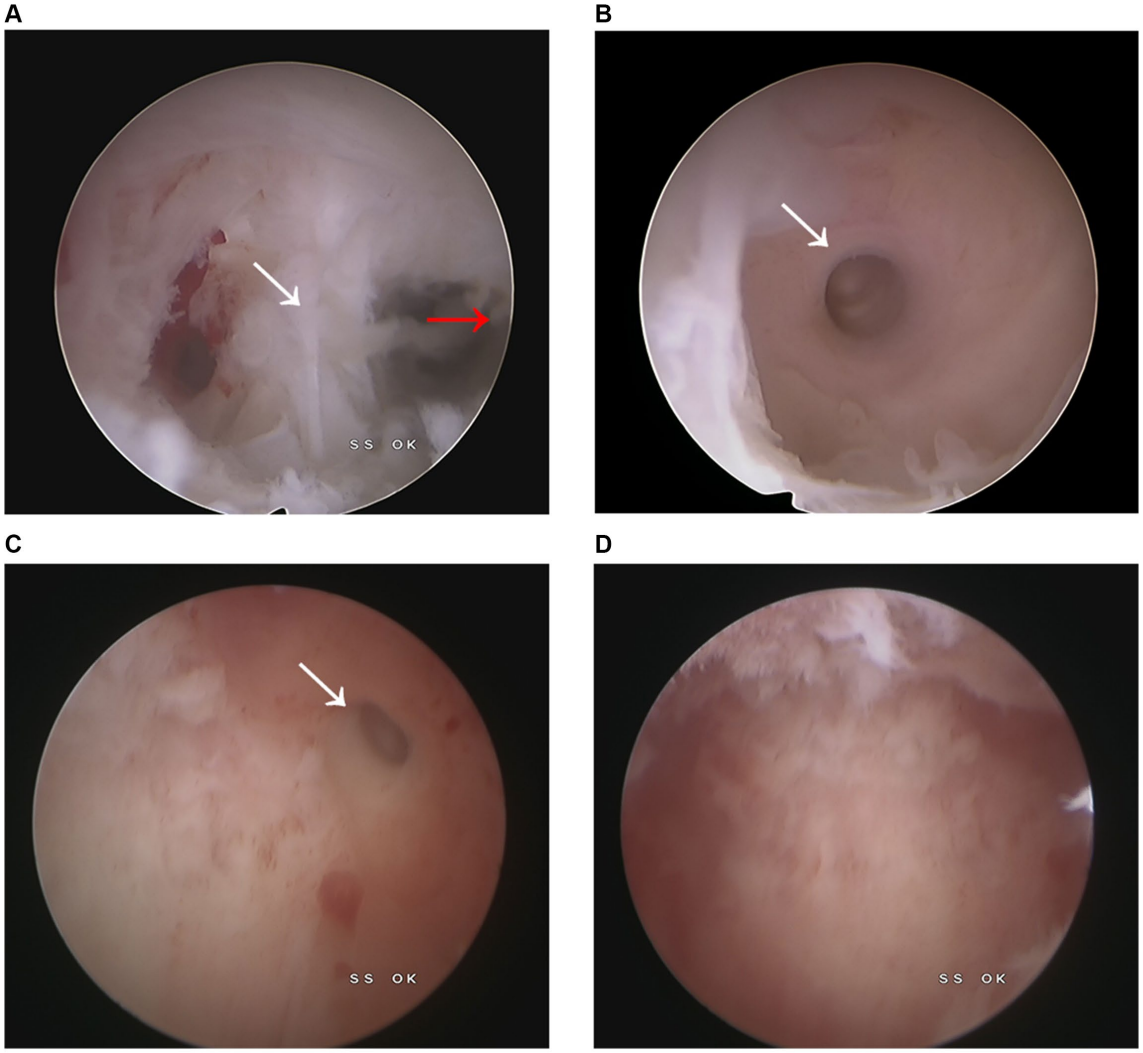
Intrauterine image. **(A)** The white arrow points to the right intrauterine adhesion, whereas the red arrow points to the oblique uterine septum. **(B)** The right fallopian tube opening could be visualized after the separation of the right uterine adhesion. **(C)** The left fallopian tube opening was exposed following the excision of the oblique uterine. **(D)** After complete resection of the oblique septum, the uterine cavity morphology was restored to normal.

**Figure 4:**
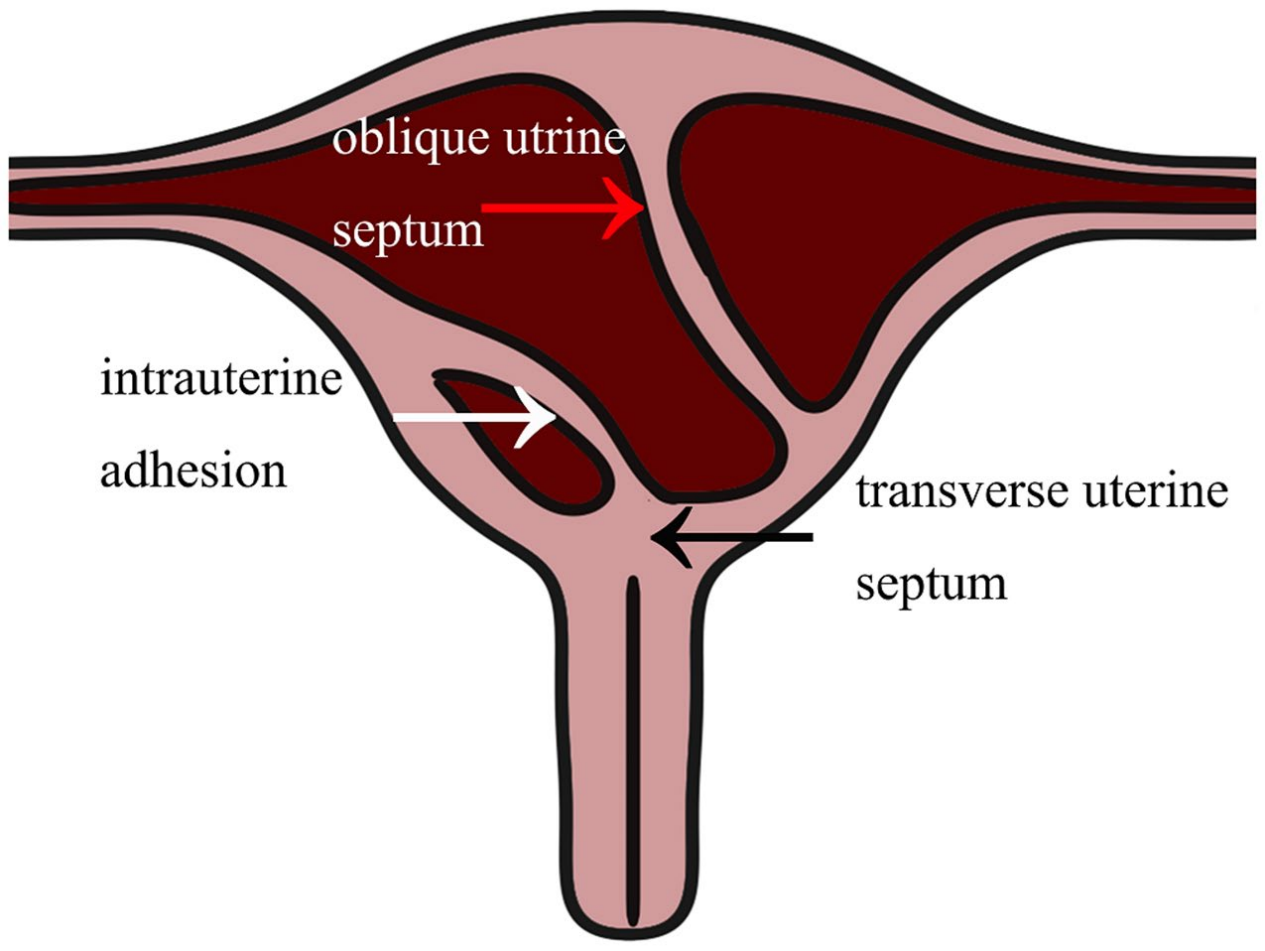
Two-dimensional model of Robert’s uterus combined with transverse uterine septum.

**Figure 5:**
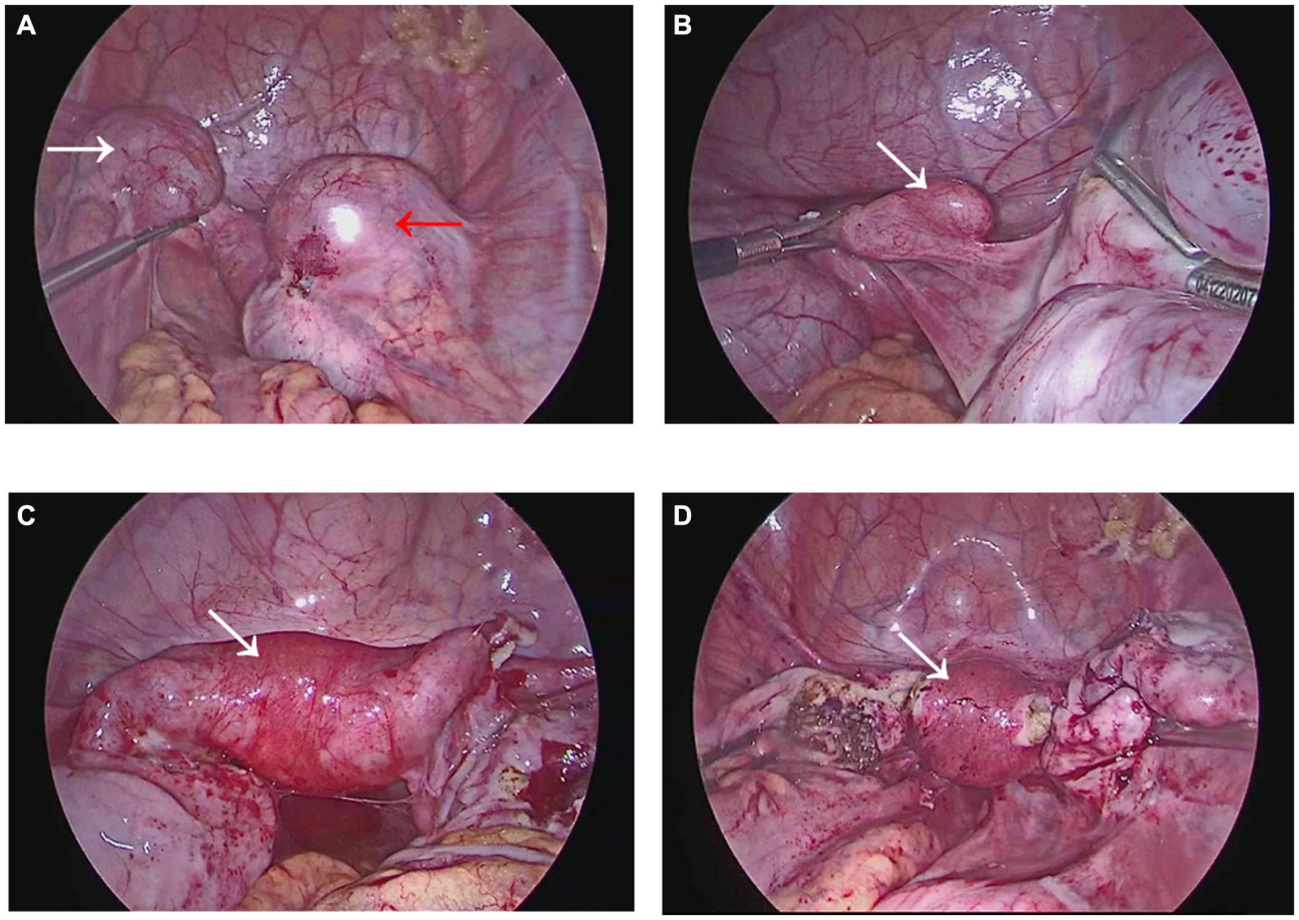
Images during laparoscopy. **(A)** The white arrow points toward the adhesion in the left attachment area, while the red arrow points toward the adhesion in the right attachment area. **(B)** Displays the blind end of the right fallopian tube. **(C)** Exhibits the external uterine silhouette prior to hysteroscopic metroplasty, with a slight downward depression near the base of the left uterus. **(D)** The external outline of the uterus after hysteroscopic metroplasty reveals a fullness at the base of the uterus, indicating that uterine morphology was restored.

During the surgical intervention, hysteroscopy (ShenDa J0100A, China) revealed a single cervix with normal cervical canal morphology. The length of the cervical canal was 4 cm, and a transverse septum was identified 7 mm above the internal opening of the cervix, preventing access to the uterine cavity Figure 2A. The septum was punctured using an transabdominal US-guided (Mindray M11T, China) probing needle, unveiling the presence of brown menstrual blood, as illustrated in Figure 2B. An oblique adhesive band was observed extending from the right side wall of the uterine cavity to the septum, and the opening of the right fallopian tube was visible, as displayed in Figures 3A,B. While the adhesive band was separated to expose the right uterine cavity, the opening of the left fallopian tube was not visible. Ultrasound showed that the left and right uterine cavities remained unconnected, and an oblique septum was found from the bottom of the left uterine wall to the left lateral wall. Miniature scissor was employed to excise the weaker portion of the septum, gradually exposing the left uterine cavity. No menstrual blood retention was found inside, and the opening of the left fallopian tube was visible (Figure 3C). Most of the endometrium was defective, with only a small amount of endometrial tissue noted at the bilateral uterine horns (Figure 3D). The two-dimensional model of the uterine cavity is portrayed in Figure 4. During laparoscopic examination, the left interstitial of the fallopian tube was thick and twisted, and a tubular structure over 3 cm long was observed at its proximal end, whereas no lumen or umbrellum structure was observed at the distal end. Besides, the left fallopian tube and left ovary were wrapped and adherent to form a mass of about 4*3 cm (Figure 5A). At the same time, the right ovary was cystically enlarged by approximately 5*5*4 cm, exhibiting a multilocular morphology and filled with clear fluid (Figure 5A). A blind end of approximately 3.5 cm long was noted in the right fallopian tube, and no umbrella structure was observed (Figure 5B). The uterus was flat in the posterior position and measured about 4*3*3 cm (Figure 5C). Following the separation of pelvic adhesion, hysteroscopic intubation was performed on the fallopian tube. The left interstitial area was blue-stained, with no methylene blue outflow observed at the distal end of the left fallopian tube. Consider that bilateral fallopian tubes may be non-functional, bilateral fallopian tubes were excised after communicating with the patient’s family. While the cystic area of the right ovary was physiological. The external outline of the uterus after hysteroscopic metroplasty reveals a fullness at the base of the uterus, indicating that uterine morphology was restored (Figure 5D). Prior to terminating the procedure, an intrauterine balloon was placed, and 4 mL of normal saline was injected. The intrauterine balloon was retained for 5 days post-operatively, and one cycle of Femoston (2 mg:10 mg) was administered to the patient.

**Figure 6:**
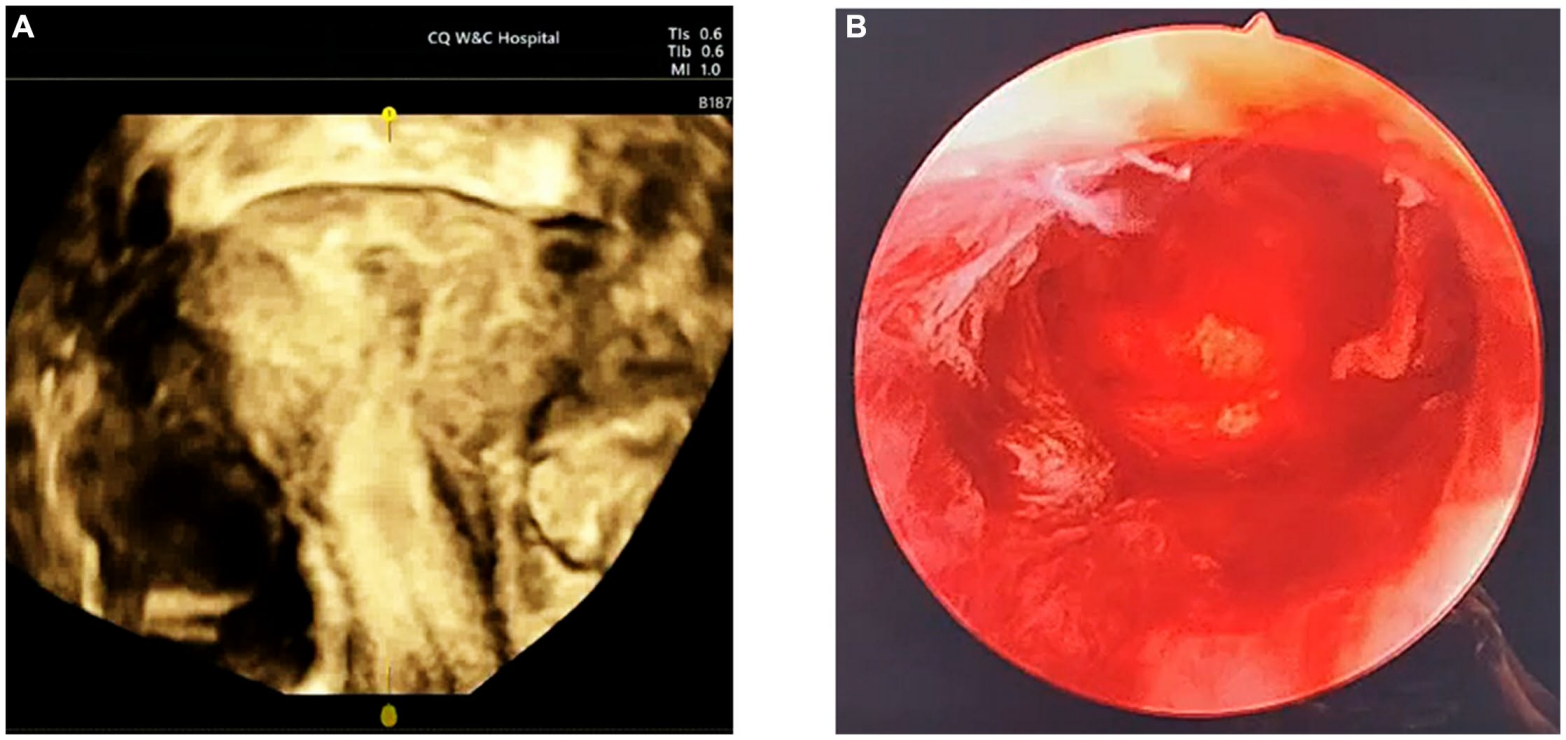
Postoperative follow-up ultrasound and hysteroscopy. **(A)** There was no significant echo in the uterine cavity by postoperative 3D ultrasound. **(B)** Follow-up hysteroscopy after menstruation showed normal uterine cavity morphology, with a thin endometrial layer visible.

On the 5th day after surgery, the intrauterine balloon was removed, and a 3D US showed no obvious abnormalities in the shape of the uterine cavity (Figure 6A). The patient menstruated 5 days after the discontinuation of Fenmorton, lasting for 3 days. On the first day, the menstrual blood soaked the area of the sanitary pad about the size of 2 coins, whereas on the 2nd and 3rd days, it was about the size of 1 coin per day. Importantly, ultrasonography exposed an endometrial thickness of about 0.3 cm, with a Y-shaped uterine cavity. Hysteroscopy conducted on the 10th day of menstruation depicted normal uterine morphology, with a thin endometrial layer in the uterine cavity, partial endometrium defects, and visible openings of the bilateral fallopian tubes (Figure 6B). At present, the patient experiences periodic menstruation with a small amount of menstrual blood. The amount and duration of menstruation were comparable to the first menstrual cycle post-operatively. Indeed, the patient did not present with dysmenorrhea.

## Discussion

The current study aimed to analyze the clinical protocol for the preoperative diagnosis and surgical management of Robert’s uterus combined with transverse uterine septum. Our clinical case provides valuable insights into the diagnosis and treatment of this unique uterine malformation.

Robert’s uterus combined with transverse uterine septum is a rare congenital malformation of the reproductive tract. Only one case of Robert’s uterus combined with a transverse uterine septum was documented in 2017 (6). The case in 2017 was roughly the same as ours, with symptoms of primary amenorrhea and cyclic pelvic pain, both of which were misdiagnosed and the surgical methods was basically the same. However, the case reported in 2017 was very simple without graphic explanation, but our case described the diagnosis and treatment process in detail and analyzed the main points. Ascribed to the rarity of this disease, it is easily misdiagnosed. Actually, we identified 46 Robert’s uterus cases in our systematic literature review, only 21 cases had a preoperative diagnosis of Robert’s uterus or an asymmetric uterine septum by US or MRI (Table 1). The majority of existing studies on Robert’s uterus comprise case reports, predominantly involving intraoperative diagnosis, while studies focused on Robert’s uterus complicated with transverse uterine septum are limited, and preoperative diagnosis proves more challenging. This patient’s uterine malformation was not diagnosed via multiple ultrasound examinations conducted in other hospitals, thereby delaying the treatment. Prolonged menstrual blood accumulation in the uterine cavity may promotes the development of endometritis and uterine adhesions. The endometrial defects impair the production of menstrual blood, which may have accounted for the alleviation of subsequent abdominal pain in patients. Even after the surgical relief of the obstruction, the endometrium defect can not be completely repaired, limiting menstrual flow and fertility. Therefore, timely detection and treatment are critical for the long-term fertility of patients.

**Table 1:** Main clinical features of Robert’s uterus cases reported in the literature.

Authors (year)	Case	Age (years)	Symptoms and key information	Live birth history	Pre-operative examination		Surgery program	Follow-up
					Ultrasonic diagnosis	MRI		
Yang H et al. 2024 (9)	1	30	Dysmenorrhea	2 cesarean sections	A septum inside the uterus and a gestational sac in the left cavity	NR	HS: removal of pregnancy LPS: hemi‑uterine excision	Complete relief of dysmenorrhea
Mahey R et al. 2023 (10)	1	13	Dysmenorrhea	-	Juvenile cystic adenomyoma	NR	LPS: hemi‑uterine excision	Complete relief of dysmenorrhea
Dong L et al. 2023 (5)	1	30	Hypomenorrhea	Diagnosed with Robert’s uterus at 22 weeks of gestation	Robert’s uterus	Robert’s uterus	Emergency cesarean delivery was performed, and the asymmetrical uterine septum was left because it ruptured completely.	NR
Panwar A et al. 2022 (11)	1	25	Smelling vaginal discharge Dysmenorrhea Abnormal uterine bleeding Dyspareunia	NR	Uterine didelphys and a right cervical collection	Bicorporeal septate uterus with septum extending up to the internal os and a single cervix	LPS HS: septal resection	Complete relief of dysmenorrhea Discharge disappeared Spontaneous conception
Liu Y et al. 2022 (12)	1	15	Dysmenorrhea accompanied by nausea, vomiting	No	Robert’s uterus	Single-horned uterus	US-guided hysteroscopic septal resection	NR
Dunphy L et al. 2022 (2)	1	40	No	1 Cesarean section	Robert’s uterus with upper lateral left horn dehiscence with significant haemoperitoneum	Upper lateral left horn dehiscence with significant haemoperitoneum	LPT: excision of the left-sided non-communicating uterine horn and the left fallopian tube	NR
Gao K et al. 2022 (13)	1	24	Hypomenorrhea Dysmenorrhea	NR	An oblique septum and fuid in the blind uterine cavity	A septum dividing the endometrial cavity into two cavities of unequal size, with hematometra within the right uterine cavity	HS: septal resection and adhesiolysis	Dysmenorrhea was disappeared gradually
Nigam A et al. 2021 (14)	1	30	Severe dysmenorrhea Spontaneous miscarriage Secondary infertility Severe deep dyspareunia chronic Pelvic pain	No	NR	Cystic adenoma or an obstructed rudimentary horn	LPS + LPT: resection of the right hemi uterus	Dysmenorrhea and chronic pelvic were significantly relieved
Ballabh S et al. 2021 (15)	4	44 18 36 21	Dysmenorrhea Constant lower abdominal which worsened during menstruation Left-sided lower abdominal pain and backache which worsened during menstruation Congestive dysmenorrhoea	No No No NR	Robert’s uterus Robert’s uterus Two uterine cavities with left haematometra Robert’s uterus	Robert’s uterus Robert’s uterus Robert’s uterus Robert’s uterus	Symptomatic treatment Surgical excision of the left obstructed hemi-uterus Lost to follow-up Excision of the left obstructed cavity with a left salpingectomy	NR
Kisu I et al. 2021 (8)	1	16	(5) Dysmenorrhea	No	Normal form of the right and left uteri, which contained blood consistent with hematometra	Right unicornuate uterus with a non-communicating left rudimentary horn	HS LPS: resection of a functioning non-communicating left rudimentary horn and salpingectomy	Dysmenorrhea was signifcantly relieved
Deenadayal M et al. 2021 (16)	5	13 25 36 39 28	Dysmenorrhea Primary infertility and dysmenorrhea Severe dysmenorrhea Severe dysmenorrhea 3 abortions at 16 weeks	No No 2 cesarean sections 2 live children	Provided no clear information A haematometra in the left hemicavity of the uterus. Normal right hemicavity and haematometra in the left hemicavity No communication between the left and right hemicavity A small communicating uterine cavity was seen on the right side, and a blind uterine cavity on the left side	A blind uterine horn with unilateral haematometra and a contralateral unicornuate uterine cavity Provided no additional information NR NR NR	LPS: endometrectomy of the blind cavity and closure of the cavity. LPS, HS: septal resection LPS: excision of the blind horn Hysterectomy with unilateralsalpingo- oophorectomy (recurrent endometrioma) Patient conceived during the investigations.	Complete relief of dysmenorrhea Complete relief of dysmenorrhea Complete relief of dysmenorrhea No pain NR
Zhang J et al. 2021 (17)	1	24	Mild dysmenorrhea	No	Complete septate or bicornuate uterus	NR	LPS US-guided HS: electrotomy of the uterine septum	Complete relief of dysmenorrhea
Liu Y et al. 2021 (18)	1	45	No	2 children via natural labor	Ectopic pregnancy in the right uterine horn	Robert’s uterus	Hysterectomy and right salpingectomy (complicated with multiple uterine fibroids and adenomyosis of the uterus were also considered)	NR
Liu Y et al. 2020 (19)	1	16	Dysmenorrhea	No	Duplex uterus and abnormal fluid collection in the right side of the uterine cavity	NR	LPS HS: asymmetric uterine septum resection	The patient successfully delivered a healthy baby two years later
Shah N et al. 2020 (20)	1	16	Severe dysmenorrhea	No	NR	Asymmetrical uterine septum, left-sided non-communicating hemicavity	LPS HS: excision of uterine septum	NR
Yang QM et al. 2019 (21)	1	23	1. Moderate dysmenorrhea 2. Chronic pelvic pain	No	Robert’s uterus with a fetal sac in the right blind cavity	Provided no additional information	LPS US-guided HS: resect the asymmetrical uterine septum and remove the pregnancy	Complete relief of dysmenorrhea
Kiyak H et al. 2017 (22)	1	15	Dysmenorrhea	No	NR	Robert’s uterus	LPS: excise the endometrial tissue of the blind cavity	NR
Mittal P et al. 2017 (23)	1	15	Severe dysmenorrhea	No	A septate uterus with heterogeneous collection in the left uterine cavity	A septate uterus with the left-sided cavity was obstructed with associated hematometra	LPT: excision of the left-sided uterine cavity and of the left adnexal endometriotic lesions	Dysmenorrhea relief
Xia E. 2015 (24)	11	16.5 (M) (15–19)	4 unmarried cases was dysmenorrhea 7 married cases was infertility 3 cases had history of enucleate choclate cyst 1 case complicated with adenomyosis	No	All cases failed in preoperative diagnosis	NR	LPS or US-guided HS: metroplasty	1 case got pregnancy 5 months after operation and cesarean section was performed at 40 + 5 gestation weeks
Ludwin A et al. 2016 (25)	1	22	Dysmenorrhea Chronic pelvic pain	NR	Robert’s uterus	NR	HS: excision of uterine septum	Complete relief of dysmenorrhea
Li J et al. 2015 (26)	1	26	Dysmenorrhea Hypomenorrhea	No	Double uteri with left-side intrauterine hemorrhage	NR	LPS HS: excision of uterine septum	The patient got pregnancy 6 months after operation and had a successful cesarean section delivery of a baby
Maddukuri SB et al. 2014 (27)	1	16	Severe cyclical lower abdominal pain during menstruation, associated with vomiting	No	Haematometra in the endometrial cavity of the uterus	Robert’s uterus	LPT: evacuation of haematometra, excision of the septum and left cervicovaginostom	NR
Vural M et al. 2011 (28)	1	24	1. Pelvic pain 2. 1 miscarriage	2 cesarean sections	Uterine anomaly was suspected	Robert’s uterus	LPT: endometrectomy was done through a hysterotomy incision and myometrium repaired	The patient got pregnancy and delivered a healthy baby by caesarean section at the 39th week of gestation.
Capito C et al. 2009 (29)	1	15	Severe abdominal and pelvic cramps	NR	A 38-mm cystic pelvic mass independent of the ovaries, which were normal	Robert’s uterus	LPT: complete endometriectomy of the blind cavity after a section of the surrounding myometrium	Complete relief
Gupta N et al. 2007 (30)	1	19	Severe dysmenorrhea Hypomenorrhea	No	Bulky uterine cavity with haematometra	Unicornuate uterus with noncommunicating functional rudimentary horn with haematometra	LPS LPT: excision of uterine septum	Complete relief
Singhal S et al. 2003 (31)	1	20	Recurrent abortion	No	Bicornuate uterus with pregnancy in the right horn, which appeared to communicate with the rest of the uterine cavity	NR	LPT: extraction of fetus, right side tubal ligation	No complaints
Alper Biler et al. 2017 (6)	1	29	1. Primary amenorrhea 2. Cyclic pelvic pain	No	Two uterine cavities and hematometra	No connection between uterus and cervix, asymmetric uterine septum	LPS US-guided HS: uterine septum was completely incised	Dysmenorrhea completely cured, regular menstruations
Di Spiezio Sardo A et al. 2016 (32)	1	30	1. Dysmenorrhea 2. Primary infertility	No	Right normal hemicavity and left hemicavity fully divided by a complete septum and not connected with the cervix	Provided no additional information	LPS HS: incision of the septum	NR
Hong XY et al. 2022 (7)	1	14	Dysmenorrhea	No	NR	Probable uterine malformation	LPS combined HS: hysteroscopic septostomy and uterine fusion	Normal menstrual volume and no dysmenorrhea

LPS, laparoscopy; Hysteroscopy, HS; LPT, laparotomy; NR, not reported.

An earlier study reported that Robert’s uterus was misdiagnosed as a unicornuate uterus with a non-communicating rudimentary horn and hematometra. The patient was then subjected to laparoscopic hemi-hysterectomy, which further lowered the probability of conception and increased the risk of uterine rupture (8). Herein, Robert’s uterus combined with the transverse uterine septum, was misdiagnosed as an incomplete septate uterus. Thus, preoperative multidisciplinary consultation and discussion are key to avoiding permanent damage to patients caused by misdiagnosis and inappropriate surgical modalities. Owing to the limitation of the MRI slice gap, MRI failed to detect the transverse uterine septum. The possibility of transverse uterine septum was proposed by ultrasound physicians during the multidisciplinary discussion. Due to the limitations of ultrasound instrument resolution and the rarity of Robert’s uterus combined with transverse uterine septum, preoperative ultrasound reports refrain from making a hasty diagnosis of transverse uterine septum. After a multidisciplinary discussion, a preliminary diagnosis of Robert’s uterus combined with transverse uterine septum was established. As anticipated, the diagnosis made through the preoperative multidisciplinary discussion was consistent with the intraoperative findings.

The surgical management of Robert’s uterus combined with transverse uterine septum is guided by some considerations. The primary objective of the treatment is to restore the physiological anatomy of the uterine cavity and enhance reproductive outcomes. Hysteroscopic metroplasty, a minimally invasive procedure, is the preferred approach for resecting the septum (33). US-guided hysteroscopic electrotomy of the uterine septum can be used for three different types of Robert’s uterus (34). However, certain factors, including the location and thickness of the septum, may necessitate alternative surgical techniques such as laparoscopic metroplasty. For this particular uterine malformation, hysteroscopic metroplasty may induce myometrial damage and uterine perforation due to mislocation, while laparoscopic surveillance may only observe the external silhouette of the uterus but cannot detect intrauterine morphology. During the operation, the adhesion was misidentified as the uterine septum. It was only under the guidance of an experienced sonographer that the real uterine septum was discovered and excised to restore the shape of the uterine cavity and avoid intraoperative missed diagnosis. Based on our experience, ultrasound-guided hysteroscopic metroplasty with the assistance of an experienced sonographer is critical to the success of the procedure. Given the limited number of cases, the effect of surgical treatment on the reproductive prognosis of patients with Robert’s uterus combined with transverse uterine septum could not be evaluated. However, the surgical alleviation of obstruction can contribute to the outflow of menstrual blood, attenuate abdominal pain, improve the quality of life of patients, and minimize the adverse effects of menstrual blood reflux on the pelvic cavity.

## Conclusion

In summary, Robert’s uterus combined with transverse uterine septum is a rare uterine malformation, with hysteroscopic metroplasty being the preferred surgical option. Due to challenges in its diagnosis, primary hospitals should actively refer patients presenting with unexplained primary amenorrhea accompanied by periodic lower abdominal pain and normal ovarian function to higher-level medical centers. Following diagnosis, surgery should be promptly performed. Preoperative multidisciplinary consultations are recommended to preliminarily assess the type of uterine malformation and the location of obstruction. Surgery should ideally be conducted under the guidance of experienced ultrasound physicians.

## Data availability statement

The original contributions presented in the study are included in the article/supplementary material, further inquiries can be directed to the corresponding authors.

## Ethics statement

The studies involving humans were approved by Ethics Committee of Women and Children’s Hospital of Chongqing Medical University. The studies were conducted in accordance with the local legislation and institutional requirements. The participants provided their written informed consent to participate in this study. Written informed consent was obtained from the individual(s) for the publication of any potentially identifiable images or data included in this article.

## Author contributions

MC: Data curation, Formal analysis, Writing – original draft, Writing – review & editing, Investigation, Visualization. XY: Investigation, Methodology, Validation, Writing – review & editing. BZ: Data curation, Software, Visualization, Writing – review & editing. JF: Data curation, Software, Visualization, Writing – review & editing. YL: Conceptualization, Investigation, Methodology, Project administration, Supervision, Writing – review & editing. YC: Conceptualization, Funding acquisition, Investigation, Methodology, Project administration, Resources, Validation, Writing – review & editing.
